# The impact of laser ablation on optical soft tissue differentiation for tissue specific laser surgery-an experimental ex vivo study

**DOI:** 10.1186/1479-5876-10-123

**Published:** 2012-06-15

**Authors:** Florian Stelzle, Ingo Terwey, Christian Knipfer, Werner Adler, Katja Tangermann-Gerk, Emeka Nkenke, Michael Schmidt

**Affiliations:** 1 Department of Oral and Maxillofacial Surgery, Friedrich-Alexander University of Erlangen-Nuremberg, Glückstrasse 11, 91054, Erlangen, Germany; 2 blz—Bavarian Laser Center, 91054, Erlangen, Germany; 3 Chair of Photonic Technologies, Friedrich-Alexander-University of Erlangen-Nuremberg, 91054, Erlangen, Germany; 4 SAOT—Graduate School in Advanced Optical Technologies, Friedrich-Alexander University of Erlangen-Nuremberg, 91054, Erlangen, Germany; 5 Department of Medical Informatics, Biometry and Epidemiology, Friedrich-Alexander University of Erlangen-Nuremberg, 91054, Erlangen, Germany

**Keywords:** Laser ablation, Laser surgery guidance, Remote optical measurement, Remote surgical methods, Spectra analysis

## Abstract

**Background:**

Optical diffuse reflectance can remotely differentiate various bio tissues. To implement this technique in an optical feedback system to guide laser surgery in a tissue-specific way, the alteration of optical tissue properties by laser ablation has to be taken into account. It was the aim of this study to evaluate the general feasibility of optical soft tissue differentiation by diffuse reflectance spectroscopy under the influence of laser ablation, comparing the tissue differentiation results before and after laser intervention.

**Methods:**

A total of 70 ex vivo tissue samples (5 tissue types) were taken from 14 bisected pig heads. Diffuse reflectance spectra were recorded before and after Er:YAG-laser ablation. The spectra were analyzed and differentiated using principal component analysis (PCA), followed by linear discriminant analysis (LDA). To assess the potential of tissue differentiation, area under the curve (AUC), sensitivity and specificity was computed for each pair of tissue types before and after laser ablation, and compared to each other.

**Results:**

Optical tissue differentiation showed good results before laser exposure (total classification error 13.51%). However, the tissue pair nerve and fat yielded lower AUC results of only 0.75. After laser ablation slightly reduced differentiation results were found with a total classification error of 16.83%. The tissue pair nerve and fat showed enhanced differentiation (AUC: 0.85). Laser ablation reduced the sensitivity in 50% and specificity in 80% of the cases of tissue pair comparison. The sensitivity of nerve–fat differentiation was enhanced by 35%.

**Conclusions:**

The observed results show the general feasibility of tissue differentiation by diffuse reflectance spectroscopy even under conditions of tissue alteration by laser ablation. The contrast enhancement for the differentiation between nerve and fat tissue after ablation is assumed to be due to laser removal of the surrounding lipid-rich nerve sheath. The results create the basis for a guidance system to control laser ablation in a tissue-specific way.

## Background

Laser surgery has emerged as an established method in advanced medicine. Laser-induced remote tissue treatment provides a number of advantages: controllable coagulation and cutting of surgical tissues with wavelength and tissue-specific cutting efficiencies [1,2]. Furthermore, laser surgery allows for a high level of sterility and precision when ablating superficial tissue [3-5]. However, the facial area in particular inherits a wealth of critically important structures and organs like nerves, salivary glands and a high number of blood vessels and laser ablation is still mainly controlled by visual feedback and therefore subjectively dependent on the surgeon. During a pulse range, it is virtually impossible for the surgeon to estimate the depth of the laser cut and identify which structure is currently being ablated. Thus, the risk of iatrogenic damage to sensitive structures like blood vessels or adjacent nerves increases dramatically [6-9]. For that reason, the application of surgical lasers is mainly limited to superficial tissue ablation. Thus, when considering profound tissue-ablation, the surgeon has to resort to a specific feedback mechanism that provides information about which structures are being affected by the laser light at the subsurface. To precisely ablate subsurface tissue and minimize the risk of iatrogenic injury, a tissue-specific feedback system based on optical tissue differentiation could provide an essential prospect. To date, various approaches have been employed for tissue differentiation by optical methods [10-13]. Optical spectroscopic techniques provide noninvasive and real-time information about the bio-morphological tissue parameters by measuring light scattering and absorption properties. In this context, diffuse reflectance spectroscopy (DRS) has proven to be a straightforward, easy-to-use and effective method for optical tissue differentiation regarding premalignant and malignant tissue differentiation [14-16]. Recently, our workgroup was able to demonstrate the prospects of diffuse reflectance spectroscopy for optical differentiation of several soft and hard tissue types [17,18].

However, when performing laser surgery, high amounts of energy are deposited in the tissue. Hence, various tissue alterations, primarily photochemical, thermal and non-linear processes, are known to occur [19-22], which may change the optical properties of tissue. Investigating the specific tissue alterations, the subsequent changes of optical properties and its impact on optical tissue differentiation present a crucial step towards implementing a remote optical feedback mechanism for laser ablation in a clinical setting. Fluorescence emission parameters were found to be altered under conditions of laser ablation [23]. The dynamic changes to tissue that occur during laser ablation were shown by optical coherence tomography [2]. Further studies were able to visualize the thermal alterations occurring during the laser ablation of cartilage, aortic and prostate tissue [24-26]. For the implementation of a remote optical feedback system for tissue-specific laser surgery, it is a major issue that even after performing laser ablation, various tissue types can be differentiated. However, there is only little information about the differentiation of physiological tissue types under laser ablation conditions using diffuse reflectance spectroscopy.

The objective of this ex vivo study was to evaluate the viability of optical tissue differentiation of physiological tissue by diffuse reflectance spectroscopy under the condition of laser surgical intervention. Additionally, the study focused on the comparison between the differentiation performance before and after laser ablation. The study placed special emphasis on the identification of nervous tissue, as preservation of these structures is essential for any surgical intervention.

## Materials and methods

### Tissue samples

5 types of tissue were obtained from bisected ex vivo pig heads (domestic pig). Types of tissue and the regions of tissue sample dissection are specified in Table 1. A total of 70 tissue samples were taken from 14 bisected pig heads – one sample each of the 5 tissue types, from each bisected head. The tissue samples had an average thickness of 5–7 mm and a dimension of 4X4 cm; besides the nervous tissue sample, which could not be obtained in this dimension due to its anatomical characteristics: nerve tissue was prepared with a length of 5 cm in total and an average diameter of 1 cm. The tissue samples were prepared with a scalpel. After dissection, the tissue samples were carefully rinsed with a sterile saline solution to remove all superficial contamination, including clotted blood particles. This step was performed very carefully in order not to mechanically alter the tissue surface with any instruments, avoiding an iatrogenic change of optical properties.

**Table 1:** Tissue samples

**tissue sample**	**region**
skin	regio buccalis
fat	regio buccalis, subcutaneal
muscle	musculus masseter
nerve	nervus infraorbitalis
mucosa	regio vestibularis

The optical measurements took place on the day of slaughter with a maximum ex vivo time of 6 h. To maintain the tissue-specific properties, desiccation was avoided by moistening the samples with a sterile saline solution and storing the tissue samples in an opaque box. All processing steps including measurements were conducted under a constant room temperature of 22°C. The animals were free of local or systemic diseases that could cause any pathological tissue alteration prior to sample extraction.

### Experimental setup

Each tissue sample was optically measured at 6 different measurement spots with a pre-defined distance between the borders of the single measurement spots of 5 mm, to avoid any bias by spot overlapping. All points of measurement were marked in the given distance to provide a standardized localization protocol, using a geometric grid that was laid over the tissue. Before and after the ablation, the same measurement points were investigated.

**Figure 1:**
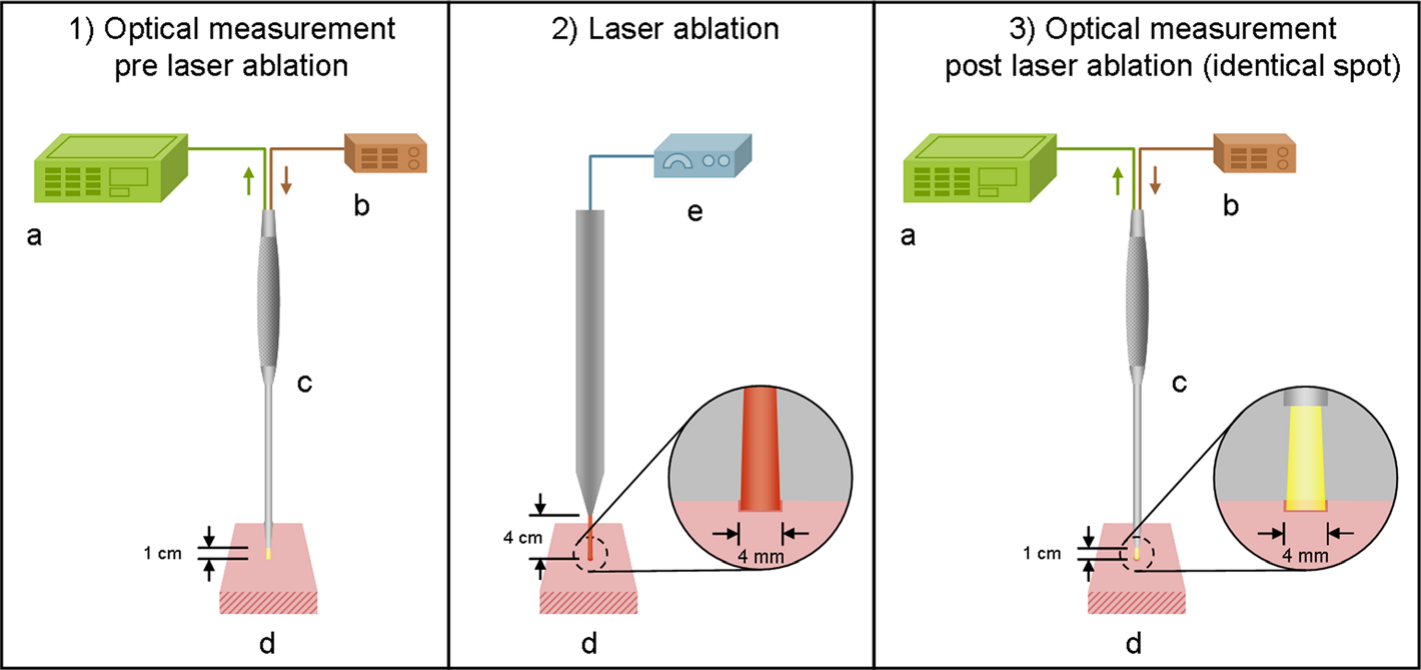
**Experimental set-up for optical measurements:** 1) Diffuse Reflectance Spectroscopy before laser ablation, 2) Tissue ablation with an Er:YAG laser (spot size ø 4 mm), 3) Diffuse Reflectance Spectroscopy in the area of ablation (**a**. Spectrometer, **b**. Pulsed xenon lamp, **c**. Reflection/backscattering probe, **d**. Tissue sample (pig, ex vivo), **e**. Er:YAG laser).

For diffuse reflectance spectroscopy of the tissues, the following experimental setup was applied: A reflection/backscattering probe (QR600-7-SR125BX, 200–1100 nm; Ocean Optics®, Dunedin, Florida, USA) was used consisting of 6 surrounding optical fibres that emit light and a central optical fibre that collects reflected light. Each fibre had a core diameter of 0,6 mm. The illuminating light was provided by a halogen lamp (HL-2000, 300–1050 nm; Ocean Optics®, Dunedin, Florida, USA). The diffuse reflection spectra were acquired by a spectrometer (QE 65000, 200–950 nm; Ocean Optics®, Dunedin, Florida, USA) combined with a computer working with the software Spectra Suite (Ocean Optics®, Dunedin, Florida, USA) (Figure 1). The following software settings were used: integration time set to 10 ms, scan to average set to 3, boxcar set to 5.

All tissue samples were placed at a distance of 1 cm to the fixed reflection/backscattering probe. The axis of the probe was aligned perpendicular to the tissue sample. After changing each measurement point, the distance between the probe and the tissue was recalibrated. During the measurement, each tissue was placed on matte black paper to avoid reflection of the underlying surface. The light spot of the probe (area of measurement) had a diameter of 4 mm. For each measurement spot (6 per tissue sample), 50 diffuse reflectance spectra were recorded sequentially—300 measurements before and 300 after ablation per tissue sample (in total, 4200 spectra before and 4200 spectra after ablation per tissue type). The experiments were performed under laboratory conditions in a dimmed environment with residual stray light. Complete darkness was avoided as these laboratory conditions would not meet the relevant practical requirements for further clinical applications.

**Figure 2:**
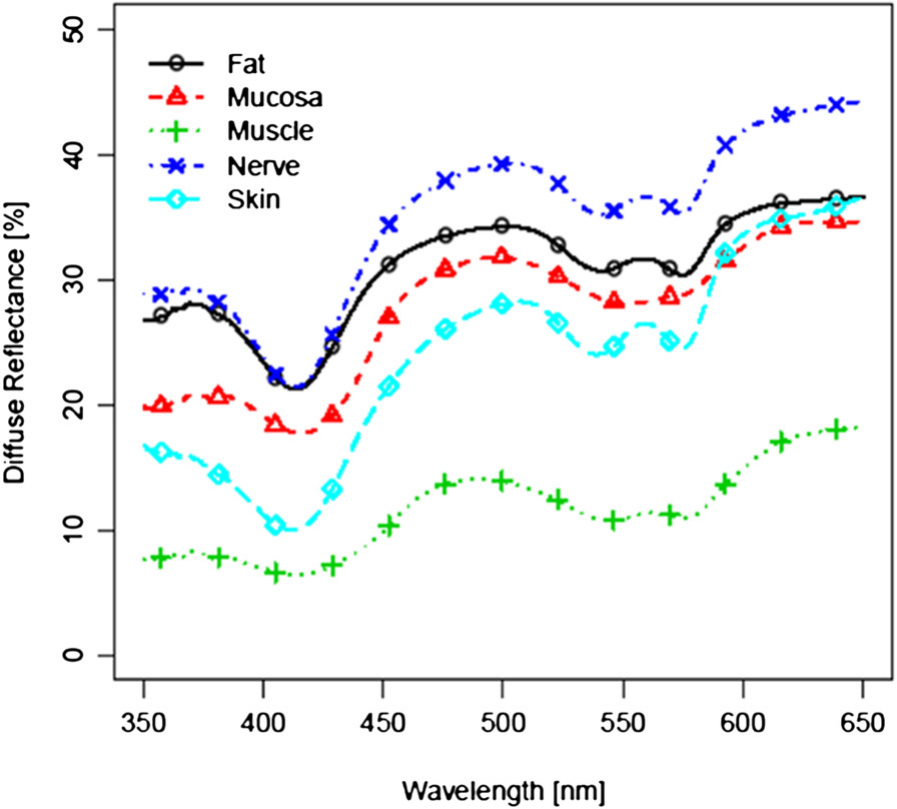
**Histological slice of ablated skin:** The ablation crater  had range of depth of 350 to 500 μm on all soft tissue types used in this study. The superficial epithelium of the skin was removed by the laser ablation uncovering sub-epithelial tissue layers. A very small darkened margin is detectable on the surface of the ablated area, indicating minimal carbonization  (Staining: H.E., magnification: 2,5x).

An Er:YAG laser (2.94 μm, Glissando, WaveLight TM, Germany) was used for tissue ablation. For each ablation, the laser emitted 30 pulses at a frequency of 10 Hz and an energy level of 500 mJ per pulse (3.97 J/cm²/pulse). The pulse duration was 350 μs. The distance chosen between the laser and the tissue was 4 cm. Using this distance, the ablation spot was fixed with a diameter of 4 mm, adapting the laser ablation crater exactly to the range of measure of the optical probe. For this purpose, the laser was fixed in a tripod (Figure 1). The ablation was carried out under constant spray water cooling with a 22°C saline solution (room temperature). With this set-up of 30 laser pulses, we were able to process all tissue samples with a constant histological ablation depth of 350 to 500 μm (Figure 2).

### Data processing

The spectra between 350 nm and 650 nm contained all relevant peaks needed for the statistical analysis. Furthermore, high noise occurred in the spectra below 350 nm and above 650 nm . This led to an exclusion of these wavelength ranges for data processing. After pre-processing, the spectra consisted of 385 data points between 350 nm and 650 nm, with a distance of 0.8 nm between the single wavelengths points. The raw signal of diffuse reflectance $S_{R_{d}}(\lambda )$ was converted into the diffuse reflectance *R*_*d*_(*λ*). The Diffuse Reflectance *R*_*d*_(*λ*) was calculated as following:

(1) $$R_{d}(\lambda )=\frac{S_{R_{d}}(\lambda )-S_{D}(\lambda )}{S_{R}(\lambda )-S_{D}(\lambda )}100\%$$

SRd(λ), Diffuse reflectance raw signal; SR(λ), Light source emission spectrum reference; SD(λ), Background signal.

The light source emission spectrum was collected as a reference spectrum, using the reflectance standard WS-1® (250–1500 nm, Ocean Optics, USA).

### Statistical analysis

We performed a principal component analysis (PCA) to reduce the number of variables used for the classification of tissue types. The PCA was applied to a data set consisting of 385 variables of 21000 centered and scaled diffuse reflectance measurements of 14 animals. Earlier work [17,18] showed that a maximum of 10 principal components was sufficient to obtain a reasonably good performance. Hence, for this study, PC 1 to 10, covering over 99% of the variability of the spectra, were chosen for further analysis. For each measurement, the probability of belonging to each of the different tissue types was estimated. To prevent overfitting, we performed subject-based cross-validation. This means that in each of fourteen runs, the probabilities of belonging to each of the tissue types were estimated for the principal components, derived from measurements of one animal. The model used for probability estimation was a multiclass linear discriminant analysis model (LDA) that was trained using principal components of all other 13 animals. Based on all tissue probabilities of all measurements, we performed an ROC analysis for all pair-wise comparisons between tissues and calculated the areas under the ROC curve (AUC) as well as the sensitivities and specificities for the optimal cut-points. The difference of classification performance before and after laser ablation was tested by comparison of the AUC values for pairwise comparisons using the DeLong test for pairwise AUC comparisons. For this statistical test, results obtained for repeated measurements were averaged prior to the comparison. The analysis was performed using data obtained before laser ablation and data obtained after laser ablation. We used the software package R V2.10.1 [27], with the packages ipred V0.8-8 [28] and Daim V1.1.0 [29] for linear discriminant analysis, and ROC analysis, respectively.

## Results

**Figure 3:**
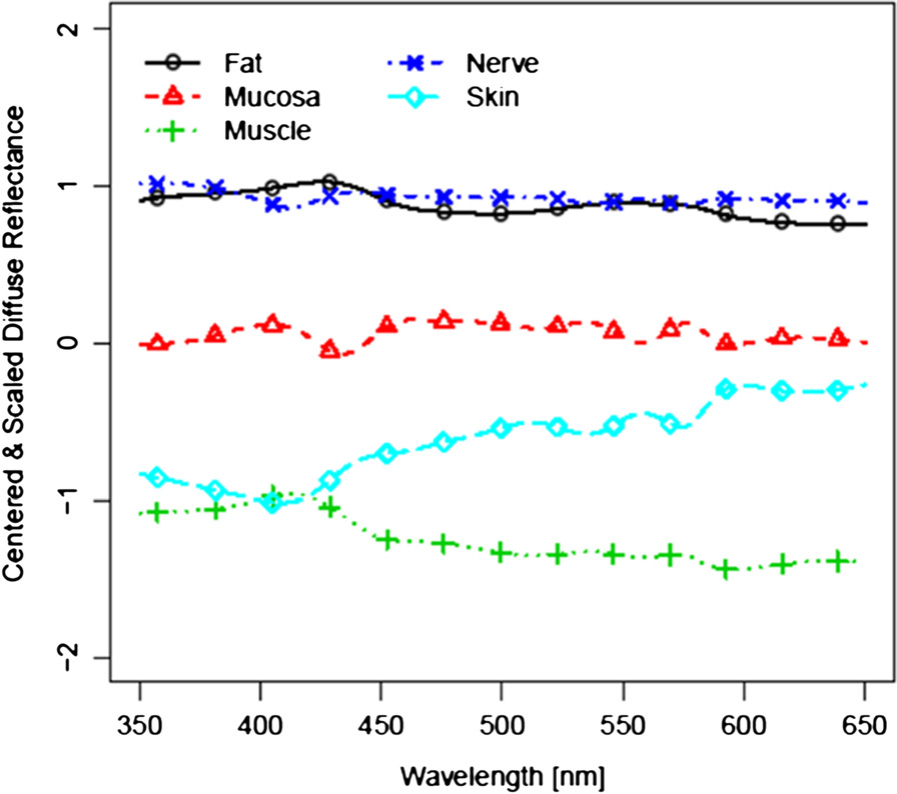
**Diffuse reflectance spectra before ablation;** Mean Standard Deviation of Measurements within the same tissue type: 7.238.

**Figure 4:**
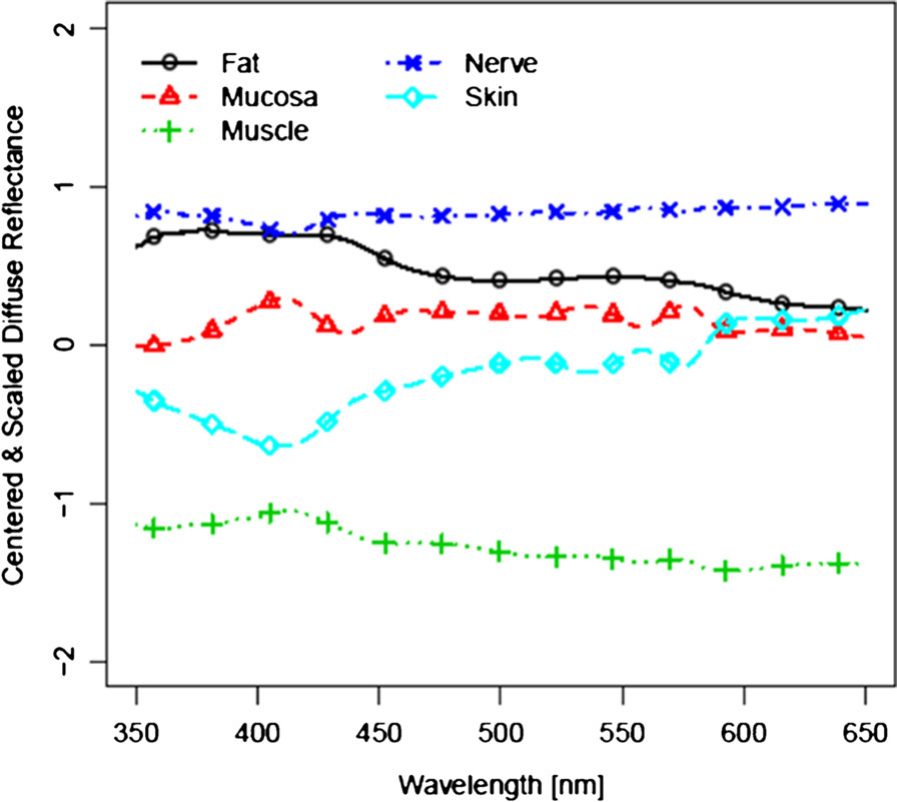
**Diffuse reflectance spectra after ablation;** Mean Standard Deviation of Measurements within the same tissue type: 7.216.

**Figure 5:**
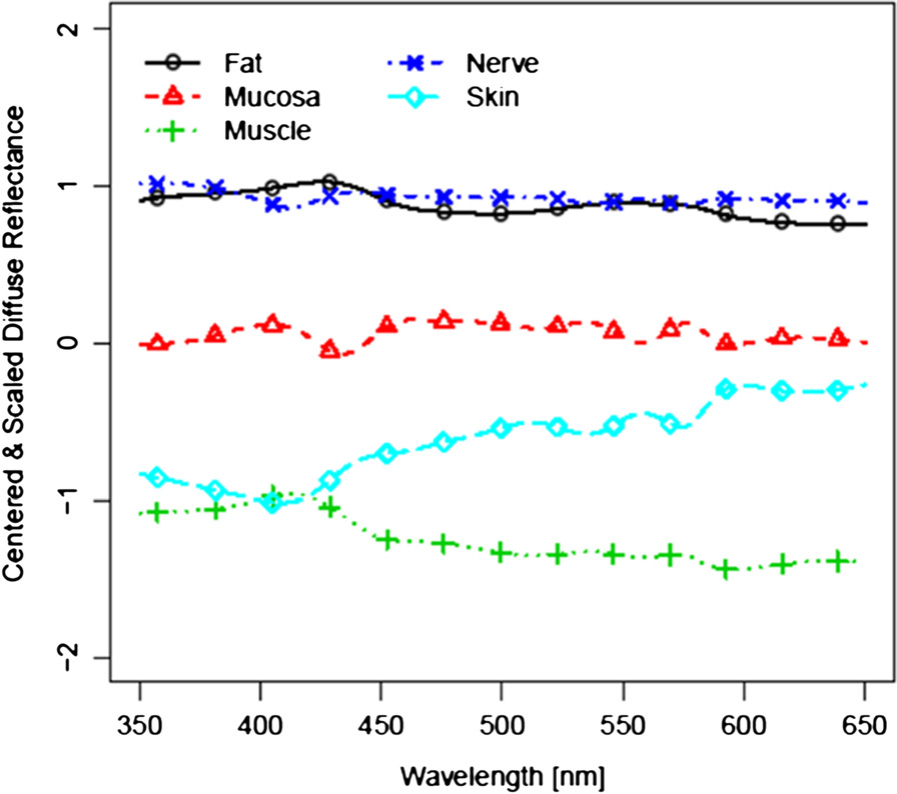
Centered and scaled diffuse reflectance spectra before ablation.

**Figure 6:**
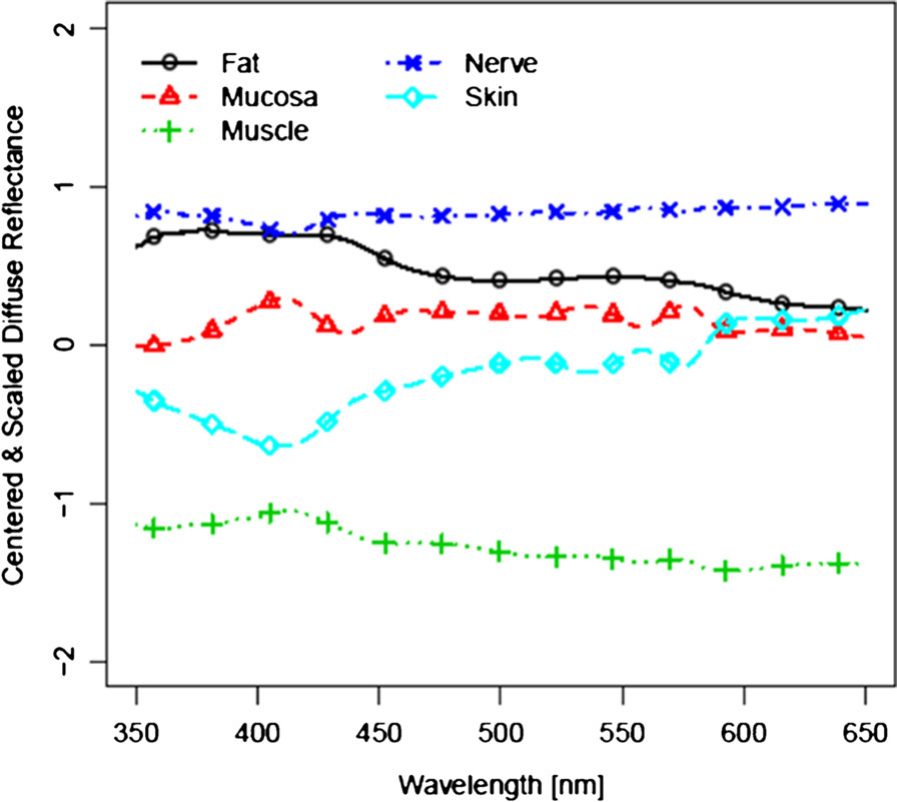
Centered and scaled diffuse reflectance spectra after ablation.

Figure 3 and 4 show the spectra for each of the five tissue types, averaged over all 14 tissue samples, before laser exposure and after ablation (i.e. 4200 spectra before and 4200 spectra after laser ablation per tissue type). For further analysis, these 4200 spectra were centered and scaled before statistical comparison (Figure 5 and 6). As the spectra turned out to be not very distinct, advanced methods of analysis, e.g. PCA followed by LDA, were used to differentiate the spectral curves. Table 2 shows the confusion matrix and the classification performance for each tissue type before and after laser ablation. Table 3 provides the area under the curve (AUC) results for each tissue type comparison before and after ablation.

**Table 2:** Confusion matrix and classification error – before and after laser ablation

**Tissue before/after**	**Classified as**					**Classification Error**
	**Fat**	**Mucosa**	**Muscle**	**Nerve**	**Skin**	
**Fat**	3094/3276	0/0	0/91	1106/831	0/2	0.263/0.22
**Mucosa**	12/0	3907/3814	241/306	40/8	0/72	0.070/0.092
**Muscle**	0/0	0/222	4200/3913	0/3	0/62	0.000/0.068
**Nerve**	1438/918	0/9	0/0	2762/2778	0/495	0.342/0.339
**Skin**	0/0	0/6	0/0	0/510	4200/3684	0.000/0.123

**Table 3:** Tissue differentiation by AUC before and after ablation

**AUC**	**Skin**	**Mucosa**	**Fat**	**Muscle**	**Nerve**
**(before/after)**					
**Skin**	-	-	-	-	-
**Mucosa**	1.00/1.00	-	-	-	-
**Fat**	1.00/1.00	1.00/1.00	-	-	-
**Muscle**	1.00/1.00	1.00/0.98	1.00/1.00	-	-
**Nerve**	1.00/0.88	1.00/1.00	0.75/0.85	1.00/1.00	-

### Results before laser ablation

Before laser ablation, a high discrimination performance was found for several tissue pairs. The mean AUC of all 10 pair-wise comparisons was 0.97. The area under the curve (AUC), as well as sensitivity and specificity for the optimal cut-point yielded results of 0.99 and higher for most of the tissue type pairs, as described in Tables 3, 4 and 5. The total classification error for single tissue type identification was 13.5% (Table 2). However, poor result were obtained for the comparison between fat and nerve (Classification error: 0.34; AUC = 0.75, sensitivity = 0.65, specificity = 0.75).

**Table 4:** Sensitivity of tissue differentiation before and after ablation

**Sensitivity**	**Skin**	**Mucosa**	**Fat**	**Muscle**	**Nerve**
**(before/after)**					
**Skin**	-	-	-	-	-
**Mucosa**	1.00/1.00	-	-	-	-
**Fat**	1.00/1.00	0.99/0.98	-	-	-
**Muscle**	1.00/1.00	1.00/0.93	1.00/0.99	-	-
**Nerve**	1.00/0.88	1.00/0.99	0.65/0.88	1.00/1.00	-

**Table 5:** Specificity of tissue differentiation before and after ablation

**Specificity**	**Skin**	**Mucosa**	**Fat**	**Muscle**	**Nerve**
**(before/after)**					
**Skin**	-	-	-	-	-
**Mucosa**	1.00/0.98	-	-	-	-
**Fat**	1.00/0.99	1.00/1.00	-	-	-
**Muscle**	1.00/0.97	1.00/0.93	1.00/0.97	-	-
**Nerve**	1.00/0.88	0.99/1.00	0.75/0.71	1.00/0.98	-

### Results after laser ablation

Regarding the results after laser ablation, the overall discrimination ability between all classes was similar to the results before laser ablation (mean AUC = 0.97). The discrimination performance for most of the tissue pairs yielded a result of over 0.99. The total classification error was 16.8% for the single tissue type identification. However, the discrimination performance between fat and nerve in particular showed lower results compared to the average performance of tissue differentiation after laser ablation (Classification error: 0.34; AUC = 0.85, sensitivity = 0.88, specificity = 0.71) (Tables 2, 3, 4 and 5).

### Comparison pre- and post- laser ablation

The comparison of the inter-class separation results before and after laser ablation yielded a varying outcome: when compared to pre-ablation conditions, a decrease in differentiation was observed in two of the tissue pairs (20%), whereas seven were found to be similar and one (10%) showed a higher AUC value. After laser ablation, the sensitivity for the optimal cut-point decreased in five of the ten tissue pair comparisons. An enhancement of sensitivity for the optimal cut-point was found after ablation in one tissue pair. The specificity for the optimal cut-point was reduced in eight tissue pairs, one turned out to be equal to pre-ablation results (Table 3 and 4). The total classification error for single tissue type identification increased (3%) (Table 2). The differentiation performance yielded a remarkable increase for the tissue pair nerve/fat: AUC results increased by 0.10, the sensitivity for the optimal cut-point by 0.23 after laser ablation. The differentiation of the tissue pair skin/nerve, however, was lowered by 0.12 as well as the sensitivity and specificity for the optimal cut-point (each by 0.12). However, when classification results for all repeated measurements were averaged, no statistically significant difference was found in AUCs before and after laser ablation.

## Discussion

The ability to automatically perform tissue differentiation is the most crucial factor for the progress of tissue-specific laser surgery. First approaches in that area showed valuable results [30-33]. Moreover, optical methods for the discrimination of tissues seem to meet the needs of a remote feedback control system, as it does not require direct contact with the tissues. Prior findings from our workgroup showed the general ability to differentiate several soft and hard tissue types ex vivo by diffuse reflectance spectroscopy [17,18]. However, performing laser ablation of biological tissues is known to cause multiple alterations, including a change of optical properties [20,34]. A successful implementation of feedback-controlled laser surgery requires the differentiation of tissues under conditions of laser ablation. Hence, it was the aim of this study to investigate the general viability of optical tissue differentiation on physiological soft tissue types by diffuse reflectance spectroscopy under conditions of laser surgical intervention.

When regarding the results before laser ablation, a high discrimination performance was found for the soft tissue pairs investigated in this study, with a mean AUC of 0.97. The average classification performance for the identification of each single tissue type in comparison to all other tissue types investigated in this study (confusion matrix) turned out to be 86.5%. These results confirm the tissue differentiation performance on non-ablated ex vivo soft tissue types, which was found in a previous study of our work group [17]. However, some differences were encountered: the AUC and sensitivity values for the tissue pair mucosa/nerve increased from 0.93 (AUC) and 0.92 (sens.) in the prior study to 1.00 (AUC&sens.) in the present study. The results of the tissue pair nerve/fat showed reduced results with an AUC of 75%, a specificity of 75%, a sensitivity of 65% and an classification error of 0.34. These parameters were found to be lower than in the prior study and are assumed to be due to the bio-morphological similarity of the two tissue types, as discussed further below [17]. However, the differences in the tissue discrimination performance between the two studies may be due to the fact that we used a different spectrometer and another data pool with different statistical parameters in this study. We currently used a spectrometer with lower resolution, performing 385 measurements in a range from 350 nm to 650 nm with an inter-measurement point distance of 0.8 nm. Another set-up was used in our prior study, consisting of a spectrometer that operated 1150 measurements in the same range with a distance of 0.26 nm, e.g., resulting in a higher resolution [17]. Moreover, the extend of the obtained data and the statistical analysis can further cause an aberration of the results. The statistical analysis in the current study was carried out based on 14 tissue samples per type of tissue, whereas in the prior study 12 tissue samples were used. Furthermore the statistical analysis, used in the current study, is based on a total of 21.000 measurements whereas in the prior study half of the data points where used (10.200 spectra). The further analysis is currently based on 10 Principal Components (prior study: 6 PCs).

Different types of lasers have been used for the purpose of tissue ablation. The excimer laser proved to allow only a low degree of tissue ablation per pulse [35,36], whereas Nd:YAG lasers, Ho:YAG lasers as well as continuous wave and long-pulsed CO2 lasers allow for a sufficiently high ablation rate performing laser surgery. However, these lasers are meant to cause a heavy thermal impact with large carbonization zones [37]. Short-pulsed (< 1 μs) CO2 lasers, ultra-short pulse lasers (Ti-Sapphire) and free running Er:YAG lasers provide sufficiently high ablation rates per pulse for rapidly processing bio-tissue as well [3,30]. Due to the fact that the tissue response is highly dependent on the wavelength of the incident light [2], the Er:YAG laser is known to be specifically suitable for fast processing of both soft and hard tissue [38,39]. Its wavelength (2.94 μm) is very close to the absorption maximum of water, the main chromophore of biological tissue, at 3 μm. The laser energy is absorbed in a very small volume of tissue, with precise removal of the irradiated tissue. It was demonstrated that Er:YAG-laser ablation comes with accurately limited lesion edges, low thermal damage, and corresponding undisturbed wound healing [40-42]. Due to these aspects the Er:YAG-laser was chosen for this study.

However, when exposing tissue to laser light, an alteration of bio-morphological properties occurs depending on wavelength, energy and irradiation time. The ablated tissue area is known to develop a carbonization zone, which scatters and absorbs incident light, followed by a zone of tissue denaturation [2]. In ER:YAG Laser systems, these undesirable thermal effects are rather small but still had a detectable depth that was reported to be ≤ 5 up to 30 μm [40-42]. Even this small area of carbonization and denaturation may cause an alteration of the optical properties, followed by a modification of the resulting diffuse reflectance spectra [43]. It is assumed that the mentioned effects of laser energy are tissue-specific. The laser impact on optical properties will vary according to the water content and the histological partition of each specific tissue type [21,44]. Additionally, the specific vascularization of each tissue type may influence the impact of laser light on tissue. Hemoglobin is known to be one of the major absorbers in biological tissue. Lukionova et al. reported an irreversible alteration of erythrocytes after exposing them to laser energy [20].

However, the influence of Er:YAG-laser ablation did not heavily alter the optical differentiation performance between the tissue pairs in this study. In general, the results after laser ablation yielded a high differentiation quality with a mean AUC of 0.97. This average value was found to be similar to the average AUC value for all tissue pairs before laser ablation. The total classification error—calculated for all tissue types of this study—was 16.8%, which yield a slightly reduced classification performance after laser ablation of 3% compared to the performance before laser ablation. More specifically, promising results were observed for the differentiation of the tissue pair skin/fat, skin/muscle, fat/muscle, muscle/nerve, mucosa/fat, mucosa/skin and mucosa/nerve, with constant differentiation qualities of 1.0 (AUC). The differentiation of these tissue pairs is meant to be of importance concerning a guided laser surgical system that will follow the anatomical tissues layer by layer. Similar findings were observed concerning the specificity of tissue differentiation. However, a slight decline of sensitivity after laser treatment was detectable for the majority of tissue pairs—but all values still ranged above 88%.

Remarkably, the differentiation parameters increased for the tissue pair nerve/fat after laser ablation: The differentiation performance rose up to 85%, the sensitivity up to 88%, compared to the results before laser ablation (75%/65%). However, the identification of nerve tissue is a crucial step concerning laser surgery guidance—heavy damage to nerve tissue was demonstrated by several studies using high energy lasers. It was reported that nerve injury by lasers may lead to major sensory and/or motor impairment, affecting the patient’s function and aesthetics [6,7,9,45,46]. As assumed in a prior work, the biological similarity of nerve and fat is followed by a reduced potential of optical differentiation [17]. Fat tissue is known to comprise large amounts of lipids like triglycerides, cholesterol and fatty acids [46]. Referring to bio-morphological criteria of nervous tissue, every nerve fiber of a peripheral nerve that was used in the current work is surrounded by a thin layer of myelin called the epineurium. In turn, each nerve fiber bundle is surrounded by another myelin sheath called the perineurium. Both of these structures consist of up to 75% lipids, e.g. 25% cholesterol, 20% galactocerebroside, 5% galactosulfatide, 50% phospholipids [46]. Hence, the tissue pair nerve/fat provides a high biological similarity, at least at the superficial layers of the samples. However, we used a constant set-up of 30 laser pulses, causing a histological ablation depth of 350 to 400 μm for all soft tissue types investigated in this study. For that reason, the surrounding myelin sheath may have been partly ablated by the laser, uncovering the bare nerve fibers. The axonal structure of nerve tissue is known to have a different biological structure compared to fat tissue, with a very low content of intracellular lipids. Hence, we assume that the laser-modified nerve structures without the surrounding myelin sheath provide a higher potential for optical differentiation, which is due to their biological diversity. On the other hand, it has to be taken into account that harming the myelin sheath already may alter nerve function and is therefore not desirable from a clinical point of view. This fact has to be considered when the results will be transferred to feedback-controlled nerve preservation during laser ablation.

An impairment of the differentiation performance was found for the tissue pair skin/nerve after laser ablation, with an AUC, a specifity and sensivity of 0.88. The underlying structure the epidermis is dominated by the connective tissue of the dermis. The epidermis and dermis of pigs provide a thickness of about 400–500 μm, followed by the subcutaneous tissue—similar to human skin [47,48]. As mentioned above, the ablation depth was found to be between 350–400 μm in this study. Hence, it is assumed that the ablation of skin removed the epidermis and exposed the underlying dermal tissue components, i.e., collagen and elastic fibres (Figure 3). Laser ablation of nerve tissue removes parts of the myelin sheath but may additionally expose the cytoskeleton of peripheral nerve tissue which is composed of protein rich neurofilaments similar to connective tissue [49]. Taking the results of the confusion matrix into account which shows that the classification error of ablated skin mainly occurred when comparing with nerve after laser ablation, it can be concluded that the optical properties of the connective tissue of the sub-epidermal tissue and the scaffold tissue of nerve show similar diffuse reflectance spectra, followed by a reduction of the differentiation performance due to their biological similarity. Considering normal body anatomy the differentiation of the tissue pair skin/nerve is not meant to be of major importance concerning a feedback system for laser guidance as major nerve branches do not run next to the skin. However, after trauma or cancer resections the anatomy will be heavily altered and the differentiation of skin and nerve may become a major issue for tissue specific laser ablation.

Compression of the tissue—when applying measurement techniques in direct contact with the tissue—is known to have an impact on optical properties in both *in vivo* and ex vivo studies [50-53]. Hence, we used remote techniques for applying the illumination light, acquiring the reflectance spectra and for laser tissue ablation, to avoid any bias by mechanical pressure on the tissue samples.

Complete darkness, which would avoid any bias from light sources others than the illumination light of the set-up, is not meant to meet the requirements of a surgical procedure on real patients. For our experiments, we have chosen a set-up with surrounding stray light to simulate an applicable environment for surgical procedures. To eliminate the influence of stray light, the diffuse reflectance spectra were adjusted by a mathematical algorithm [54].

In the current preliminary investigation we performed a total of 300 measurements per tissue type for each of the 14 tissue samples in order to show the general feasibility of tissue identification and differentiation by this method and further gain a data pool for each of the 5 tissue types. For the clinical *in vivo* implementation in a feedback system for laser surgery, it is necessary to establish a greater data pool as a base for tissue identification. Then, a minimal number of spectra can be recorded after each laser pulse to identify the tissue type using the trained LDA after the transformation dictated by the PCA.

The promising results of this study have to be considered with care concerning some limitations: First, the study was conducted on pigs’ tissue. Interspecies differences, e.g. human/pig, may show varying results when transferring this method to other animal models or humans. Second, ex vivo tissue is similar but not identical to *in vivo* tissue due to its decreasing moisture and blood content, the missing blood circulation and its progressing de-oxygenation of hemoglobin [55,56]. Thus, further research is necessary to transfer the technique to *in vivo* tissue, taking into account the influence of circulation and oxygenation. Third, the ablation was performed with an Er:YAG-laser, which is known to cause minimal alterations to the surrounding tissue. As any laser interaction with biological tissue depends considerably on the wavelength, the results of this study may not be transferable to other laser types. Even though, this study demonstrated the general viability of tissue differentiation under the influence of laser ablation by diffuse reflectance spectroscopy.

## Conclusion

The results of this ex vivo study yield an overall high differentiation potential for various soft tissue types after Er:YAG laser ablation, performing diffuse reflectance spectroscopy followed by PCA and LDA. In general, a similarly high differentiation quality with a total classification errors of 13.51% before laser ablation and 16.83% after laser ablation was found. However, Er:YAG laser exposure of the tissue slightly reduced the sensitivity and specificity for the optimal cutpoint for some tissue pairs, but still yielded results of more than 85%. For the tissue pair nerve/fat, the differentiation quality and sensitivity was even enhanced by laser treatment. Further investigations have to be conducted to prove how the results obtained in the current study can be transferred to an *in vivo* application. The results of this study set the base for an automated optical guidance for tissue-specific laser surgery under the influence of laser ablation.

## Ethics approval

Not necessary. The experimental study was carried out on tissues that were provided by a slaughterhouse.

## Competing interests

The authors declare that they have no competing interests

## Authors’ contributions

FS, IT, CK and KTG carried out the tissue preparation as well as the optical measurements. IT, MS and KTG installed and adapted the optical set-up. WA participated in the design of the study and performed the statistical analysis. FS, CK, EN and MS performed the data analysis and assessment. FS and MS conceived of the study, participated in its design and coordination and drafted the manuscript. All authors read and approved the final manuscript.
